# A mass spectrometry-based method for comprehensive quantitative determination of post-transcriptional RNA modifications: the complete chemical structure of *Schizosaccharomyces pombe* ribosomal RNAs

**DOI:** 10.1093/nar/gkv560

**Published:** 2015-10-10

**Authors:** Masato Taoka, Yuko Nobe, Masayuki Hori, Aiko Takeuchi, Shunpei Masaki, Yoshio Yamauchi, Hiroshi Nakayama, Nobuhiro Takahashi, Toshiaki Isobe

**Affiliations:** 1 Department of Chemistry, Graduate School of Science and Engineering, Tokyo Metropolitan University, Minami-osawa 1-1, Hachioji-shi, Tokyo 192-0397, Japan; 2 Core Research for Evolutional Science and Technology (CREST), Japan Science and Technology Agency (JST), Sanbancho 5, Chiyoda-ku, Tokyo 102-0075, Japan; 3 Biomolecular Characterization Team, RIKEN Center for SustainableResource Science, 2-1 Hirosawa, Wako, Saitama 351-0198, Japan; 4 Department of Biotechnology, United Graduate School of Agriculture, Tokyo University of Agriculture and Technology, Saiwai-cho 3-5-8, Fuchu-shi, Tokyo 183-8509, Japan

## Abstract

We present a liquid chromatography–mass spectrometry (LC-MS)-based method for comprehensive quantitative identification of post-transcriptional modifications (PTMs) of RNA. We incorporated an in vitro-transcribed, heavy isotope-labeled reference RNA into a sample RNA solution, digested the mixture with a number of RNases and detected the post-transcriptionally modified oligonucleotides quantitatively based on shifts in retention time and the MS signal in subsequent LC-MS. This allowed the determination and quantitation of all PTMs in *Schizosaccharomyces pombe* ribosomal (r)RNAs and generated the first complete PTM maps of eukaryotic rRNAs at single-nucleotide resolution. There were 122 modified sites, most of which appear to locate at the interface of ribosomal subunits where translation takes place. We also identified PTMs at specific locations in rRNAs that were altered in response to growth conditions of yeast cells, suggesting that the cells coordinately regulate the modification levels of RNA.

## INTRODUCTION

To date, >100 different types of PTMs have been found in various types of cellular RNAs, including transfer (t)RNA, rRNA, small nuclear RNA, small nucleolar (sno)RNA and messenger (m)RNA (1). PTMs in tRNA have roles in the formation and stabilization of proper tertiary structure and maintenance of the gene decoding system (2,3), and those in rRNA in the stabilization of local ribosome structure, regulation of ribosome biogenesis and translational activity and modulation of antibiotic drug resistance (4). In higher organisms, dysregulation of RNA PTMs can cause severe pathological consequences, such as human cancer, neurological disorders, metabolic diseases, viral infections and autoimmune disorders (5–10). Thus, the accumulation of knowledge about RNA PTMs will have an impact on biology and medicine.

Analysis of RNA PTMs has relied for decades on thin-layer chromatography (11) or biochemical techniques (12–14), which have limitations related to speed, sensitivity and specificity. Although the recent development of sequencing-based techniques has solved many of these problems and now allows for sensitive, transcriptome-wide PTM analysis (15–21), those methods have relatively poor quantitative capacity and their application is restricted to specific PTMs. A number of MS-based approaches have also been developed for direct determination of the sites of PTMs within RNA (22–25). Those analytical strategies are typically based on RNase mapping, in which PTMs are identified by the mass shifts of oligonucleotides within an RNase-digested sample relative to the theoretical masses predicted from the gene or cDNA sequence. Although this type of method can effectively detect PTMs within relatively small RNAs (26), it still requires a conventional reverse transcription assay for comprehensive PTM analysis of large RNAs (27). More recently, Waghmare *et al*. (28) and Popova *et al*. (29) described a method for MS-based PTM analysis using an isotope-labeled RNA synthesized *in vivo* and defined dozens of PTMs in 16S and 23S *E. coli* rRNAs. Although this method is not applicable for a comprehensive PTM analysis, it is notable in that the method can measure—in a manner similar to metabolic isotope labeling in proteomics (30)—the relative abundance of modified nucleotides within cells in different physiological states. The method described here, which we call Stable Isotope-Labeled riboNucleic Acid as an internal Standard (SILNAS), is an extension of our studies of MS-based RNA analytical techniques (31,32) and allows comprehensive identification and quantification of PTMs by using a stable isotope-labeled RNA synthesized *in vitro*. To our knowledge, this is the first report that describes a method to directly estimate the absolute stoichiometry of RNA PTMs and that presents the complete PTM map of rRNAs from a eukaryote.

## MATERIALS AND METHODS

### Chemicals

Standard laboratory chemicals were obtained from Wako Pure Chemical Industries. Sodium guanosine-^13^C_10_ 5′-triphosphate (98 atom% ^13^C) and RNase A were obtained from Sigma-Aldrich. Sodium cytidine-^13^C_9_ 5′-triphosphate and sodium uridine-^13^C_9_ 5′-triphosphate were purchased from Santa Cruz Biotechnology, Inc. RNase T1 was purchased from Worthington and further purified by reversed-phase LC. Triethylammonium acetate buffer (pH 7.0) was purchased from Glen Research. Chemically synthesized oligonucleotides that mimic *S. pombe* 5S rRNA (positions 90–115) with or without modifications of ribose or base molecules were obtained from JBioS. The chemical structures of these synthetic oligonucleotides are given in Supplementary Table S1.

### Strains and media

HeLa cells (American Type Culture Collection) were cultured in DMEM (Sigma-Aldrich) supplemented with 10% fetal bovine serum (Hana-Nesco Bio) and 100 U/ml penicillin-streptomycin (Invitrogen) in a humidified atmosphere containing 5% CO_2_ at 37°C, and the medium was changed every 2 or 3 days.

Wild-type SP6 *Schizosaccharomyces pombe* (American Type Culture Collection) was used throughout this study. The cells were grown in YE medium (0.5% yeast extract, 3% glucose, 300 mg/l leucine; Wako Pure Chemical Industries) at 30°C and harvested during logarithmic growth phase (∼1.0 × 10^7^/ml) as determined by counting cells in culture.

### Preparation of cellular rRNAs

Total RNAs were prepared from HeLa cells or yeast cells using the TRIzol reagent (Invitrogen Life Technologies). To obtain 5S, 5.8S or 18S rRNA, total RNAs were applied to a reversed-phase LC system (LC-20A, Shimadzu) equipped with a capillary PLRP-S 4000 column (2-mm i.d. × 100 mm in length, 8-μm particle size; Polymer Laboratories), eluted with a 60-min linear gradient from 11.6% to 14% acetonitrile in 100 mM triethylammonium acetate (pH 7.0) including 0.1 mM ammonium phosphate dibasic at a flow rate of 50 μl/min at 60°C and fractionated by monitoring ultraviolet absorption at 260 nm (33). Only rRNAs with >95% purity were used.

### *In vitro* transcription of internal standard RNAs

To construct the plasmids for *in vitro* transcription of our internal standard RNAs, the DNA of human 5.8S rRNA and *S. pombe* 5S, 5.8S, 18S and 25S rRNA was amplified by polymerase chain reaction (PCR) from genomic DNA. The primers used for the PCR are given in Supplementary Table S1. The amplified DNA of each rRNA was inserted into the XhoI/EcoRI, XhoI/HindIII, KpnI/HindIII or HindIII/NotI sites of plasmid pBluescript II KS (Agilent Technologies, Inc.). Before *in vitro* transcription, the plasmid was linearized with SpeI to terminate the product at the end of the rRNA. To synthesize RNA, 2 μg of template DNA was incubated and transcribed using a Megascript T3 kit (Invitrogen). When RNA was synthesized, guanosine-^13^C_10_ 5′-triphosphate, cytidine-^13^C_9_ 5′-triphosphate or uridine-^13^C_9_ 5′-triphosphate solution was used instead of the respective 5′-triphosphate reagent that contained carbons with a natural isotope distribution. The RNA was precipitated in ethanol, solubilized in nuclease-free water and then purified further by reversed-phase LC as described above.

### RNase H cleavage of rRNAs

Sequence-specific RNase H cleavage was performed using a guide DNA to target the RNA site (34). DNA molecules used herein are presented in Supplementary Table S1. Fission yeast 18S rRNA (2 pmol) was digested with 15 U of RNase H (Takara Bio Inc.) at 42°C for 1 h in the presence of a synthetic 20- to 28-mer guide DNA (4 pmol) complementary to the cleavage site in 100 μl of 40 mM Tris-HCl (pH 7.7), 4 mM MgCl_2_ and 1 mM DTT. Before RNase H digestion, the sample RNA was denatured at 65°C for 10 min. The RNase H digests were directly loaded onto reversed-phase LC columns described above to purify the resulting RNA fragments.

### LC-MS apparatus for RNA analysis

The LC system used was as described (32,35). The column was prepared with a fused-silica capillary (150-μm i.d. x 120 mm in length) packed with a reversed-phase material (Develosil C30-UG-3, 3-μm particle size; Nomura Chemical). LC was performed at a flow rate of 100 nl/min using a 60-min linear gradient from 10% to 32% methanol in 10 mM triethylammonium acetate (pH 7.0). The eluate was introduced into an LTQ-Orbitrap hybrid mass spectrometer (Thermo Fisher Scientific) through an electrospray ion source. The mass spectrometer was operated in a negative mode, switching automatically between Orbitrap-MS and linear ion trap–MS/MS acquisition as described (32).

### Database search and interpretation of RNA MS/MS spectra

We used the software Ariadne (31) for database searches and assignments of MS/MS RNA signals by utilizing the *S. pombe* genome database (http://www.pombase.org/downloads/datasets). We used the following search parameters: the maximum number of missed cleavages was set at 1; the variable modification parameters included two methylations per RNA fragment for any residue; RNA mass tolerance of 20 ppm and MS/MS tolerance of 750 ppm were allowed. When Ariadne identified a methylated oligonucleotide, the MS/MS spectrum was manually inspected to distinguish the methyl group attached to the base or sugar by the presence or absence of methylated base loss from the parent ion. Where necessary, the position of methyl group on the nucleotide base was predicted based on the structure of *Saccharomyces* *cerevisiae* rRNA (http://people.biochem.umass.edu/fournierlab/snornadb/main.php) (36).

### Cyanoethylation of pseudouridine by acrylonitrile

RNA samples were digested before the derivatization reaction. The digests were cyanoethylated by the method developed by Mengel-Jorgensen *et al*. (37), with minor modifications. Briefly, 34 μl of 40% ethanol/1.2 M triethylammonium acetate (pH 8.6) and 1 μl acrylonitrile were added to 5 μl of RNase T1-digested RNAs (<1 pmol), and the resulting mixture was incubated at 70°C for 120 min. After cyanoethylation, the reaction mixture was diluted with 10 volumes of water and analyzed by LC-MS. The optimal conditions for cyanoethylation were determined by test reactions using a standard RNA (yeast tRNA^Phe^, R4018; Sigma-Aldrich) with various acrylonitrile amounts and ethanol percentages and for different incubation times. Under the optimized conditions determined (2.5% acrylonitrile, 35% ethanol, 70°C), about 30% of the pseudouridine and 5% of the uridine molecules were cyanoethylated (data not shown).

### Additional procedures for RNA analysis

Denaturing polyacrylamide gel electrophoresis and in-solution RNase T1 and RNase A digestion of RNA were performed as described (35).

## RESULTS AND DISCUSSION

**Figure 1.**
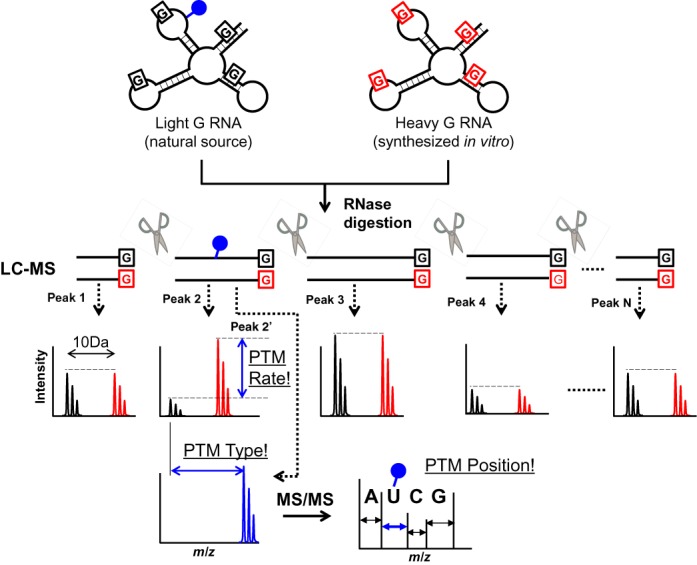
Schematic of SILNAS. RNA from natural sources or cells grown in ^12^C-containing guanosine (light-G RNA) is mixed with an equal amount of synthetic RNA that mimics the sample RNA but was transcribed *in vitro* with ^13^C_10_-labeled guanosine (shown by red Gs; heavy-G RNA). The mix is digested and analyzed by LC-MS. The sample RNA fragment without PTM elutes in a single peak with the corresponding heavy G-containing fragment as paired MS signals of an equivalent signal height at 10 Da apart, whereas a modified oligonucleotide elutes at a different chromatographic position from the corresponding heavy fragment and shows a distinct MS spectrum characteristic of each PTM. In SILNAS, the site of modification can be assigned comprehensively by searching the chromatographic peak containing only the light fragment, and the rate of PTM can be estimated quantitatively by comparing the signal heights of light and heavy fragments. The PTM site is determined by concurrent MS/MS analysis. Closed blue circle, a chemical group introduced by PTM.

SILNAS is based on the fact that (i) an *in vitro*-transcribed RNA does not carry PTMs and (ii) RNA PTMs generally cause a shift in the retention time (Rt) and MS signal in LC-MS. Our strategy is depicted and described in Figure 1. With this method, all RNA fragments without modified nucleotides exhibit single chromatographic peaks containing the ‘light’ and ‘heavy’ RNA fragments with identical signal heights, whereas a light fragment carrying modified nucleotides appears in a separate peak with a different chromatographic Rt relative to the corresponding heavy fragment derived from the reference RNA. In this way, we can account for all PTMs, even those found within long runs of RNA, by searching for the light fragments and can identify the type and the position of the PTM from its MS and MS/MS spectra. SILNAS also provides quantitative information about PTMs so that we can estimate the stoichiometry of the PTM at each modification site by comparing the signal heights of the unmodified light and heavy RNA fragments (Figure 1). We estimated the accuracy of SILNAS-based quantification by measuring the synthetic light and heavy RNA fragments and found that the ratio of heavy/light in the MS signal parallels the molar ratio of the mixture (*R*^2^ > 0.99) with an average standard deviation of <5% (Supplementary Figure S1). SILNAS can also be used to determine the site of pseudouridylation, a ‘mass-silent’ modification found abundantly in many types of RNA, by the shift of retention time in LC or by coupling the cyanoethylation with acrylonitrile to discriminate pseudouridine (Ψ) from uridine (see Methods and Supplementary Figure S2).

**Figure 2.**
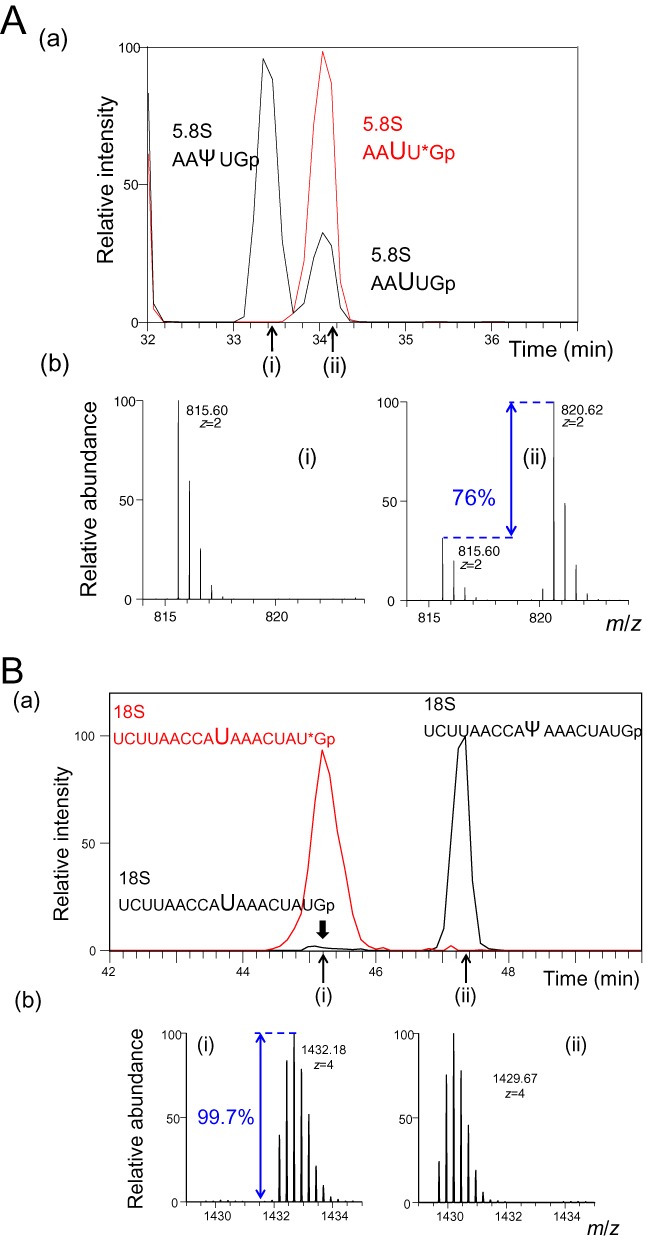
SILNAS-based PTM analysis. (**A**) Ψ76 in *S. pombe* 5.8S rRNA. (a) Extracted ion monitoring of RNase T1 fragments of 5.8S rRNA containing PTMs. Because PTM of U76 was incomplete (76% as estimated by SILNAS), RNase T1 produced two fragments, ^74^AAΨU^78^Gp and ^74^AAUU^78^Gp, from the sequence region 74–78 of 5.8S rRNA. In a subsequent LC analysis, the fragment ^74^AAΨU^78^Gp eluted earlier than the unmodified fragment ^74^AAUU^78^Gp and the corresponding heavy fragment ^74^AAUU^78^*Gp (*G, 13C10-guanosine) derived from the reference RNA. Oligonucleotide ion masses: [AAUUGp]^2−^ and [AAΨUGp]^2−^ (black line), *m*/*z* 815.60; [AAUU*Gp]^2−^ (red line), *m*/*z* 820.62. (b) MS spectrum for each RNA peak, (i) and (ii). From the signal heights of MS spectra of the light and heavy AAUUGp ion, the extent of PTM was estimated as 76%. (**B**) Ψ1039 in *S. pombe* 18S rRNA. (a) Extracted ion monitoring of RNase T1 fragments of 18S rRNA containing PTMs. Although PTM of U1039 was almost complete (99.7% as estimated by SILNAS), a trace amount of unmodified RNase T1 fragment, ^1030^UCUUAACCAUAAACUAU^1047^Gp, was detected with the modified fragment ^1030^UCUUAACCAΨAAACUAU^1047^Gp from the sequence region 1030–1047 of 18S rRNA. In a mass chromatogram, the modified fragment ^1030^UCUUAACCAΨAAACUAU^1047^Gp eluted later than the unmodified fragment ^1030^UCUUAACCAUAAACUAU^1047^Gp and the corresponding heavy fragment ^1030^UCUUAACCAUAAACUAU^1047^*Gp (*G, ^13^C_10_-guanosine) derived from the reference RNA. Oligonucleotide ion masses: [UCUUAACCAUAAACUAUGp]^4−^ and [UCUUAACCAΨAAACUAUGp]^4−^ (black line), m/z 1429.67; [UCUUAACCAUAAACUAU*Gp]^4−^ (red line), m/z 1432.18. The mass windows used for extraction were 15 ppm. (b) MS spectrum for each RNA peak, (i) and (ii). From the signal heights of MS spectra of the light and heavy UCUUAACCAUAAACUAUGp ion, the extent of PTM was estimated as 99.7%.

To validate SILNAS, we analyzed PTMs in human 5.8S rRNA, a 157-nucleotide RNA containing two methylated nucleotides and two Ψs (Um14, Ψ55, Ψ69 and Gm75) (38) and were able to identify all of the PTMs (Supplementary Table S2). Importantly, SILNAS defined the stoichiometry of the PTMs. For instance, the guanosine at position 75 in ^71^AAUUGmCA^78^Gp was 92% methylated (Supplementary Figure S3 and Supplementary Table S2). The SILNAS-based quantitative analysis actually provided the levels of modification at all of the PTM sites in *S. pombe* rRNAs (described below). Figure 2 illustrates the extracted ion chromatograms of Ψ76 and Ψ1039 in *S. pombe* 5.8S and 18S rRNA, respectively.

To prove the utility of SILNAS, we applied this method to the comprehensive PTM analysis of rRNAs of a eukaryotic model organism, *Schizosaccharomyces pombe*; i.e. 5S, 5.8S, 18S and 25S rRNA consisting of 119, 160, 1842 and 3498 nucleotides, respectively. We easily ascertained the complete chemical structures of the 5S and 5.8S rRNAs and found no PTMs in 5S rRNA and only a single Ψ at position 76 in the 5.8S rRNA (shown in Figure 2A). For the 18S and 25S rRNAs, we performed extensive analyses of the RNA fragments produced by RNase T1 digestion and, where necessary, carried out complementary analysis for the RNA fragments produced by RNase A digestion of a reference 18S rRNA labeled with ^13^C-CTP and ^13^C-UTP to obtain overlapping fragments having 3′ cytidine or uridine to align the RNase T1 fragments. In addition, when long RNA segments produced multiple nucleolytic fragments having the same sequence, the RNA was systematically segmented with RNase H (34) to avoid the production of such fragments and to distinguish the redundant sequences (see Methods and Supplementary Figure S4). Thus, we could align all the fragments and determine the complete chemical structure of these 1842- and 3498-nucleotide eukaryotic rRNAs carrying a variety of PTMs (Supplementary Figures S5 and S6).

The *S. pombe* rRNAs contained 122 sites of modification: 47 in small subunit (SSU) and 75 in large subunit (LSU) rRNA (one in 5.8S and 74 in 25S rRNA). SSU rRNA contained 23 sites of pseudouridylation, 19 sites of 2′-O-methylation, 2 sites of base acetylation, 2 sites of base dimethylation, a single site of base monomethylation and a single site of 1-methyl-3-(3-amino-3-carboxypropyl)pseudouridylation (Supplementary Figure S7), and LSU rRNA contained 32 sites of pseudouridylation, 37 sites of 2′-O-methylation and 6 sites of base monomethylation (Supplementary Table S3 and Supplementary Figures S5 and S6). Our analysis also assigned two acetylcytidines at positions 1297 and 1815 in 18S rRNA. For more than three decades, it has been reported that 18S rRNA from a broad range of eukaryotic cells, from budding yeast to mammals, has two acetylcytidines (39,40) but those sites of acetylation have only recently been determined (41). That study also identified the *N*-acetyltransferase responsible for this cytidine acetylation and showed that the *S. pombe* strain in which it was mutated has a slow-growth phenotype and is defective in forming the 18S rRNA from the precursor RNA (41).

**Figure 3.**
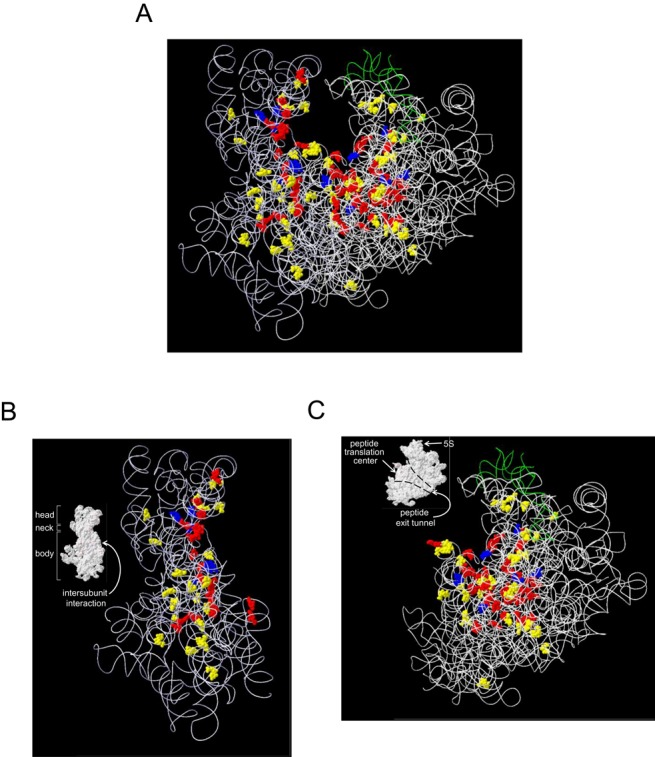
Three-dimensional PTM map of the yeast ribosome. The PTMs in *S. pombe* rRNAs were assigned to the three-dimensional structure of *S. cerevisiae* rRNAs (3U5B.pdb and 3U5D.pdb). The RNA backbone is expressed as a ribbon structure: 18S, 5.8S and 25S rRNA, white; 5S rRNA, green. The PTMs within the SSU and LSU are colored as follows: pseudouridines, yellow; 2′-O-methylations, red; base modifications, blue. (**A**) SSU and LSU. (**B**) SSU. (**C**) LSU. Inset B and C, The surface structure of ribosomal subunits. The domain name is indicated by white letters with an arrow.

Because a high-resolution 3D structure of the *S. pombe* ribosome is not available, we assigned our identified modified nucleotides to the 3D structural map of the *S. cerevisiae* ribosome. The rRNAs of both yeast species exhibit high sequence similarity (>85% identity), and we could easily map all the modified nucleotides on *S. cerevisiae* rRNAs. By surveying the resulting 3D modification map, we readily recognized that most (∼92%, 112/122; Supplementary Table S3) modified nucleotides were located in functionally important interior regions of the ribosome, including the peptidyl transferase center (PTC); the A, P and E sites of tRNA and mRNA binding; the polypeptide exit tunnel and the interacting surfaces of both the SSU and LSU (Supplementary Table S3 and Figure 3A). In the SSU, the sites of PTM are clustered within a plane that includes the head, neck and body regions (Figure 3B), although the PTMs are distributed uniformly on the three lobules of its secondary structure (Supplementary Figure S8A). In contrast, the sites of PTMs in the LSU accumulated in lobule II, IV and V among six secondary lobules that form a monolithic structure (Supplementary Figure S8B), and almost all the PTMs clustered around the PTC and subsequent peptide exit tunnel, which is shaped like a funnel (Figure 3C). PTMs are essentially absent from the external regions and periphery of the interior surface regions, which are mainly covered by ribosomal proteins (Supplementary Figure S9). Thus, the complete 3D modification map of the *S. pombe* ribosome confirmed a global landscape of modified nucleotides within the eukaryotic ribosome, as predicted in previous studies based on partial PTM data (reviewed by Decatur and Fourniel (42)).

**Figure 4.**
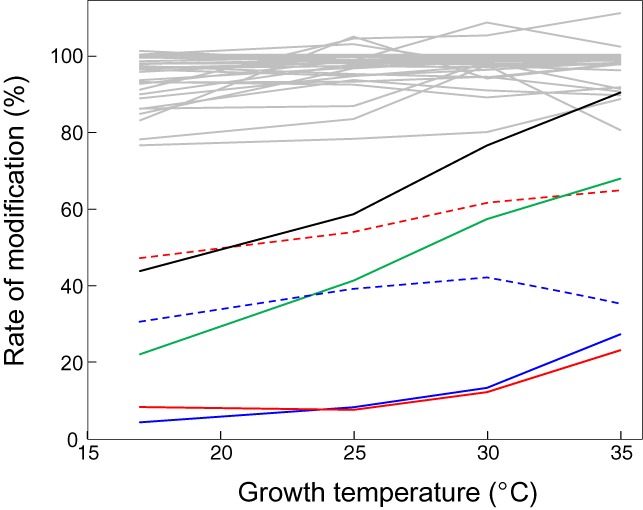
Growth temperature-dependent changes in the stoichiometry of PTMs. Rates of typical PTMs in *S. pombe* rRNAs are shown: Ψ76 in 5.8S rRNA (black solid line) and Ψ208 (red solid line), Ψ305 (blue dotted line), Ψ909 (blue solid line), Ψ1231 (green solid line) and Ψ1435 (red dotted line) in 18S rRNA. Gray lines show changes in the stoichiometry of 34 other PTM sites in 18S rRNA. Detailed quantitative data with standard deviations of the measurement (from four or eight independent replicates) are presented in Supplementary Table S3.

The SILNAS-based quantitative analysis of the stoichiometry of PTM at each modification site showed that the extent of PTM is site specific. Namely, most of the 122 PTM sites in the *S. pombe* rRNAs were almost fully modified (>85%), whereas the extent of modification at several sites was ∼10–60% (Supplementary Table S3). Of particular interest is that the extent of modification of specific nucleotides was significantly affected by the growth conditions of the yeast cells from which the RNA was extracted. We actually estimated the quantitative levels of ∼40 modification sites in 5.8S and 18S rRNAs of S. pombe cultivated under different growth temperatures, including Ψ, Am, Cm, Um, Gm, ac^4^C, m^7^G, m^6^_2_A and m^1^acp^3^Ψ in 5.8S and 18S rRNA. Among those, we found that the modification levels of several Ψs varied with growth temperature to the significant extent, while the levels of most others were nearly 100% regardless of growth temperature (Supplementary Table S3). Namely, the pseudouridylation at U76 in 5.8S rRNA increased from 44% to 90% in response to raising the growth temperature from 17°C to 35°C. Likewise, the baseline pseudouridylation at U1231 in 18S rRNA was 22% at 17°C and increased to 68% at 35°C (Figure 4). Although the roles of these nucleotides are not clear, we assume that the pseudouridylation at specific nucleotides in rRNA may serve regulatory roles in ribosome biogenesis and/or function. This would be relevant to the recent finding that pseudouridylation occurs at specific sites of particular mRNAs as a part of the dynamic regulatory process of cellular function in response to environmental signals (19–21).

In this study, we described an MS-based method, SILNAS, for comprehensive PTM analysis of RNA and presented its application to construct the first complete PTM map of eukaryotic rRNAs. We expect that it will serve as a useful resource for studies of the structure, function and biogenesis of the ribosome. SILNAS compares with previous methods (28,29) that use a metabolically labeled reference RNA; however, these technologies have different characteristics, both in utility and practice (Table 1), and SILNAS has an advantage for high-throughput, comprehensive identification of PTMs in a wide range of RNAs. We expect that the unique capability of SILNAS to determine the stoichiometry of PTMs opens up the possibility of studying the cellular dynamics of RNA PTMs and will help us to address questions that require information about the global landscape of RNA modifications within cells.

**Table 1.** SILNAS *versus* Metabolic Labeling

	SILNAS	Metabolic labeling^a^	Explanation/comment
Method of Isotope labeling	genetic engeneering-based *in vitro* labeling	cell culture-based *in vivo* (metabolic) labeling	
PTM in a reference RNA	no	yes	Metabolically labeled RNA undergoes PTMs in the cell
Detection of PTM	data dependent	prediction of MS shift reqired	
Detection of pseudouridine	shift of retention time in LC/chemical modification, e.g. cyanoethylation	metabolic labeling in the presence of 5,6-D_2_ uridine	
Quantitation of PTM	absolute quantitation (to estimate the absolute stoichimetry of PTM )	relative quantitaion (to estimate the relative changes in PTM)	
Comprehensive PTM analysis	possible	difficult	*In vivo* labeling method cannot detect "unknown" PTMs with unpredictable MS shifts

^a^According to the references (28) and (29).

## SUPPLEMENTARY DATA

Supplementary Data are available at NAR Online.

SUPPLEMENTARY DATA
