# Supplementary material for: A mass spectrometry-based method for comprehensive quantitative determination of post-transcriptional RNA modifications: the complete chemical structure of Schizosaccharomyces pombe ribosomal RNAs
Source: Nucleic Acids Res. 2015 Oct 10;43(18):e115. doi: 10.1093/nar/gkv560 (PMC4605285; doi:10.1093/nar/gkv560)
Supplement: SUPPLEMENTARY DATA [file supp_43_18_e115__index.html]

A mass spectrometry-based method for comprehensive quantitative determination of post-transcriptional RNA modifications: the complete chemical structure of Schizosaccharomyces pombe ribosomal RNAs — SUPPLEMENTARY DATA 

# A mass spectrometry-based method for comprehensive quantitative determination of post-transcriptional RNA modifications: the complete chemical structure of *Schizosaccharomyces pombe* ribosomal RNAs

## SUPPLEMENTARY DATA

- SUPPLEMENTARY DATA
- SUPPLEMENTARY DATA
