# Supplementary material for: A mass spectrometry-based method for comprehensive quantitative determination of post-transcriptional RNA modifications: the complete chemical structure of Schizosaccharomyces pombe ribosomal RNAs
Source: Nucleic Acids Res. 2015 Oct 10;43(18):e115. doi: 10.1093/nar/gkv560 (PMC4605285; doi:10.1093/nar/gkv560)

Supplementary Information for

A mass spectrometry–based method for comprehensive quantitative determination of post-transcriptional RNA modifications: the complete chemical structure of *Schizosaccharomyces pombe* ribosomal RNAs

Masato Taoka*[1,2], Yuko Nobe[1,2], Masayuki Hori[1], Aiko Takeuchi[1], Shunpei Masaki[1,2], Yoshio Yamauchi[1,2], Hiroshi Nakayama[2,3], Nobuhiro Takahashi[2,4], Toshiaki Isobe*[1,2]

[1]Department of Chemistry, Graduate School of Science and Engineering, Tokyo Metropolitan University, Minami-osawa 1-1, Hachioji-shi, Tokyo 192-0397, Japan

[2]Core Research for Evolutional Science and Technology (CREST), Japan Science and Technology Agency (JST), Sanbancho 5, Chiyoda-ku, Tokyo 102-0075, Japan

[3]Biomolecular Characterization Team, RIKEN Advanced Science Institute, 2-1 Hirosawa, Wako, Saitama 351-0198, Japan

[4]Department of Biotechnology, United Graduate School of Agriculture, Tokyo University of Agriculture and Technology, Saiwai-cho 3-5-8, Fuchu-shi, Tokyo 183-8509, Japan

* To whom correspondence should be addressed:
Masato Taoka, mango@tmu.ac.jp

SUPPLEMENTARY FIGURE LEGENDS

Supplementary Figure 1. Calibration curves of SILNAS-based quantification for specific PTMs. Calibration curves were generated for the synthetic RNA fragments containing the PTM (a) AAΨCCGp and (b) ACAACmAUGp. Data points represent the mean of three independent replicates.

Supplementary Figure 2. SILNAS-based determination of pseudouridine after cyanoethylation. (a) Pseudouridine-containing oligonucleotide ions extracted from LC-MS chromatograms before and after cyanoethylation. In this example, an RNA fragment (AAΨCCGp) eluted very closely to the equivalent heavy G–containing fragment (AAUCC*Gp) derived from the reference RNA in LC (left). After cyanoethylation, however, the cyanoethyl-pseudouridine–containing fragment AAcyeΨCCGp eluted much earlier than the byproduct of the cyanoethylation reaction,

AAcyeUCCGp, which contained cyanoethyl-uridine (right). The mass of each oligonucleotide ion was as follows: $[AA\Psi CCGp]^{2-}$, *m*/*z* 967.628; $[AAUCC*Gp]^{2-}$ (red line), *m*/*z* 972.644; $[AAcye\Psi CCGp]^{2-}$, *m*/*z* 994.141; $[AAcyeUCC*Gp]^{2-}$ (red line), *m*/*z* 999.158 (Ψ, pseudouridine; cye, cyanoethylated nucleotide; *G, $^{13}C_{10}$-guanosine). The mass windows used for extraction were 15 ppm. (b) Tandem mass spectrum of cyanoethylated AAcyeΨCCGp. The cyanoethylated RNA ion (arrowhead in a) was analyzed by collision-induced dissociation. The position of pseudouridine was identified by manual interpretation of the a-, c-, w- and y-type product ion series as indicated in the figure. The series ions assigned from the spectrum are indicated on the RNA sequence in the inset.

Supplementary Figure 3. SILNAS-based PTM analysis of methylated guanosine at position 75 (Gm75) in human 5.8S rRNA. (a) Extracted ion monitoring of RNase T1 fragments of 5.8S rRNA containing a PTM. 2′-O-Methylation of G75 inhibited the activity of RNase T1 and generated the fragment ^{71}AAUUGmCA^{78}Gp (92% modified) as well as the unmodified, RNase T1–sensitive fragment ^{71}AAUU^{75}Gp. Oligonucleotide ion masses: $[AAUUGp]^{2-}$ (black line), *m*/*z* 815.60; $[AAUU*Gp]^{2-}$ (red line), *m*/*z* 820.62; $[AAUUGmCAGp]^{2-}$ (green line), *m*/*z* 1312.18 (Gm, 2’-*O*-methyl guanosine). (b) The mass spectra of RNA ions at the retention times indicated by (i) and (ii) in a. The extent of 2′-O-methylation in Gm75 was estimated to be 92% based on the signal heights of $[AAUUGp]^{2-}$ and $[AAUU*Gp]^{2-}$. The site of 2′-O-methylation was determined by the MS/MS spectrum of $[AAUUGmCAGp]^{2-}$ (data not shown).

Supplementary Figure 4. An example of digestion and purification of RNase H fragment from rRNA.

(a) Strategy to produce Fragment H11 (blue arrow, 560 bases, see also Supplementary Figure 6) from *S. pombe* 25S rRNA. The 25S rRNA (open square) was mixed with an equal amount of *in vitro* transcribed heavy isotope-labeled reference RNA. The mixture was added with the synthesized DNA, p25S-1201-dna and p25S-1774-dna (See Supplementary Table 1), which was complementary to the position 1201-1225 and 1774-1799 of 25S rRNA (closed squares) respectively, and digested with RNase H.

(b) Agarose gel electrophoresis profile of the RNase H digested 25S rRNA. Purified 25S rRNA with (+) and without (-) RNase H were analyzed on 1.0% agarose gel and stained with SYBR Gold. The RNA size markers are indicated on the left. Fragment H11 was detected around 560 bases.

(c) Purification of 25S Fragment H11 by reversed phase LC. The RNase H digest mixed

with *in vivo*-transcribed reference RNA and *in vitro*-transcribed 25S rRNAs were applied to a PLRP-S 4000 column (See Experimental section). Sequence covered by the fragment was confirmed by LC-MS analysis coupled with Ariadne search.

Supplementary Figure 5. The complete PTM map of *S. pombe* 18S rRNA and the fragments used for the structural analysis. Black solid bars denote the fragments produced by RNase T1 digestion of the whole 18S rRNA molecule, and black shaded bars denote those produced by RNase A digestion. Blue arrows are RNase H–digested fragments of the 18S rRNA (fragment numbers correspond to mapping data shown in Supplementary Table 3). Blue solid bars denote RNase T1–digested fragments obtained from the RNase H fragment, and blue shaded bars indicate RNase A–digested fragments. All fragments were identified by the Ariadne search program (see online Methods) except several heavily modified RNA fragments, which were identified by manual inspection of their MS/MS spectra. Modified RNAs are shown in blue letters, and their abbreviations are according to Modomics (*Nucleic Acids Res.* 2013; 41:D262-7; http://modomics.genesilico.pl/): B, 2'-*O*-methylcytidine; :, 2'-*O*-methyladenosine; #, 2'-*O*-methylguanosine; J, 2'-*O*-methyluridine; P, pseudouridine; M, N^4-acetylcytidine; α, 1-methyl-3-(3-amino-3-carboxypropyl)pseudouridine; 7, 7-methylguanosine; ζ, N^6,N^6-dimethyladenosine.

Supplementary Figure 6. The complete PTM map of *S. pombe* 25S rRNA and the fragments used for the structural analysis. Symbols used in the figure are given in the legend to Supplementary Figure 5. Red solid bars denote the fragments produced by RNase H digestion after RNase T1 digestion of the whole 25S rRNA molecule. The RNase H digestion was performed with the guide oligonucleotide p25S-1063-chimera or p25S-1718-chimera (Supplementary Table 1).

Supplementary Figure 7. SILNAS-based PTM analysis of m1acp3Ψ1208 in *S. pombe* 18S rRNA.

(a) Extracted ion monitoring of RNase T1 fragments of 18S rRNA containing PTMs. Because PTM of U1208 was complete (100% as estimated by SILNAS), RNase T1 only produced a modified fragment, ^{1206}ACm1acp3ΨCAACAC^{1215}Gp from the sequence region 1206-1215 of 18S rRNA. In a subsequent LC analysis, this fragment eluted earlier than the unmodified heavy fragment ^{1206}ACUCAACAC^{1215}*Gp derived from the reference RNA. Note that the figure also shows the RNase T1 fragments of sequence

isomer, ^{1172}CACCACAAU^{1181}Gp and ^{1172}CACCACAAU^{1181}*Gp, derived from 18S rRNA and its reference RNA, which were co-eluted in a single peak just before the fragment ^{1206}ACm1acp3ΨCAACAC^{1215}Gp. Oligonucleotide ion masses: [CACCACAAUGp]$^{3-}$/[ACUCAACACGp]$^{3-}$(black line), m/z 1067.48; [ACm1acp3ΨCAACACGp]$^{-}$ (blue line), m/z 1105.83 (m1acp3Ψ, 1-methyl-3-(3-amino-3-carboxypropyl)-pseudouridine); [ACUCAACAC*Gp]$^{3-}$(red line), m/z 1070.82 (*G, $^{13}C_{10}$-guanosine). The mass windows used for extraction were 15 ppm. (b) The MS spectra for RNA peak (i), (ii) and (iii), are shown. From the signal heights of MS spectra of the light and heavy ACUCAACACGp ion, the extent of PTM was estimated as 100%. (c) Tandem mass spectrum of ACm1acp3ΨCAACACGp. The ion (arrowhead in a) was analyzed by collision-induced dissociation. The position of m1acp3Ψ was identified by manual interpretation of the a-, c-, w- and y-type product ion series as indicated in the figure. The series ions assigned from the spectrum are indicated on the RNA sequence in the inset.

Supplementary Figure 8. The PTMs on SSU and LSU rRNA of *S. pombe*. The figure was drawn according to the 3D source file of *S. cerevisiae* rRNA (3U5B.pdb and 3U5D.pdb) with the assistance of Assemble2 software. The local structure specific for the LSU of *S. pombe* was calculated with Centroidfold (http://www.ncrna.org/centroidfold/) and then added to the figure. Each modified residue is shown as a symbol within a circle. Stoichiometry of the modified residue is indicated with a doughnut graph. RNA domains are identified with Roman numerals. Black balls, adenine; blue balls, uridine; red balls, guanine; green balls, cytosine. (A) Secondary structure of SSU rRNA of *S. pombe*. (B) Secondary structure of LSU rRNA of *S. pombe*. The figure contains 25S, 5.8S and 5S rRNAs.

Supplementary Figure 9. PTMs in yeast rRNA with and without ribosomal proteins. The PTMs in *S. pombe* rRNA were assigned to the three-dimensional structure of *S. cerevisiae* rRNA (3U5B.pdb and 3U5D.pdb) as in Figure 3. The ribosomal proteins were depicted according to the 3D source file of *S. cerevisiae* (3U5C.pdb and 3U5E.pdb). (A) SSU and (B) LSU. The bare RNA structure is shown on the left, and the RNA structure in the presence of ribosomal proteins is shown on the right.

Supplementary Figure 1

(a)

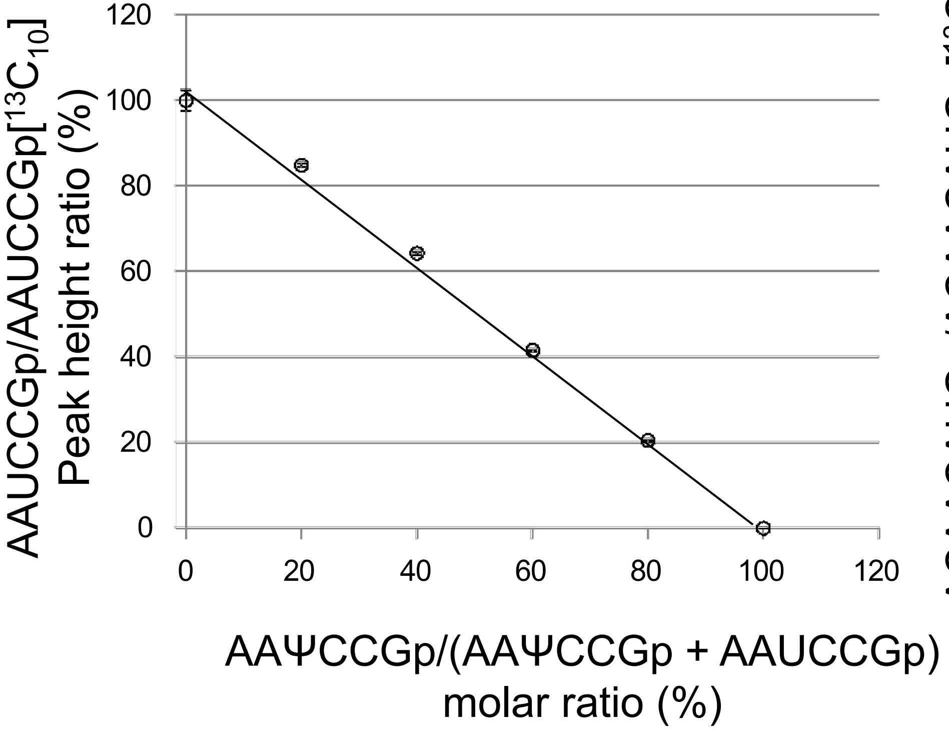

(b)

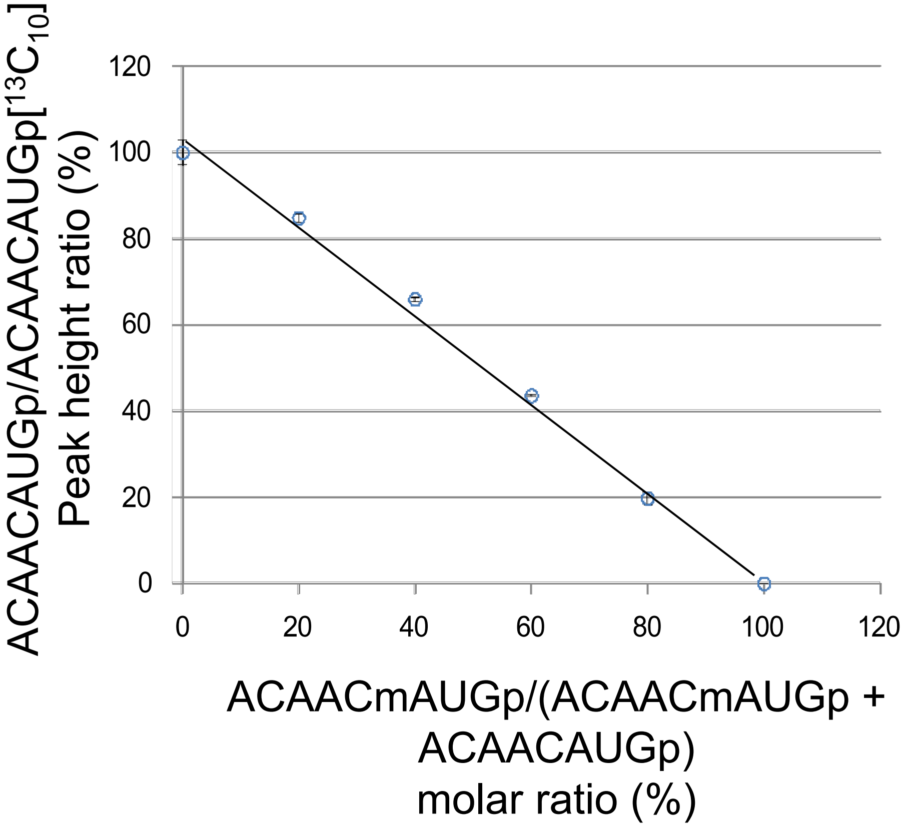

Supplementary Figure 2

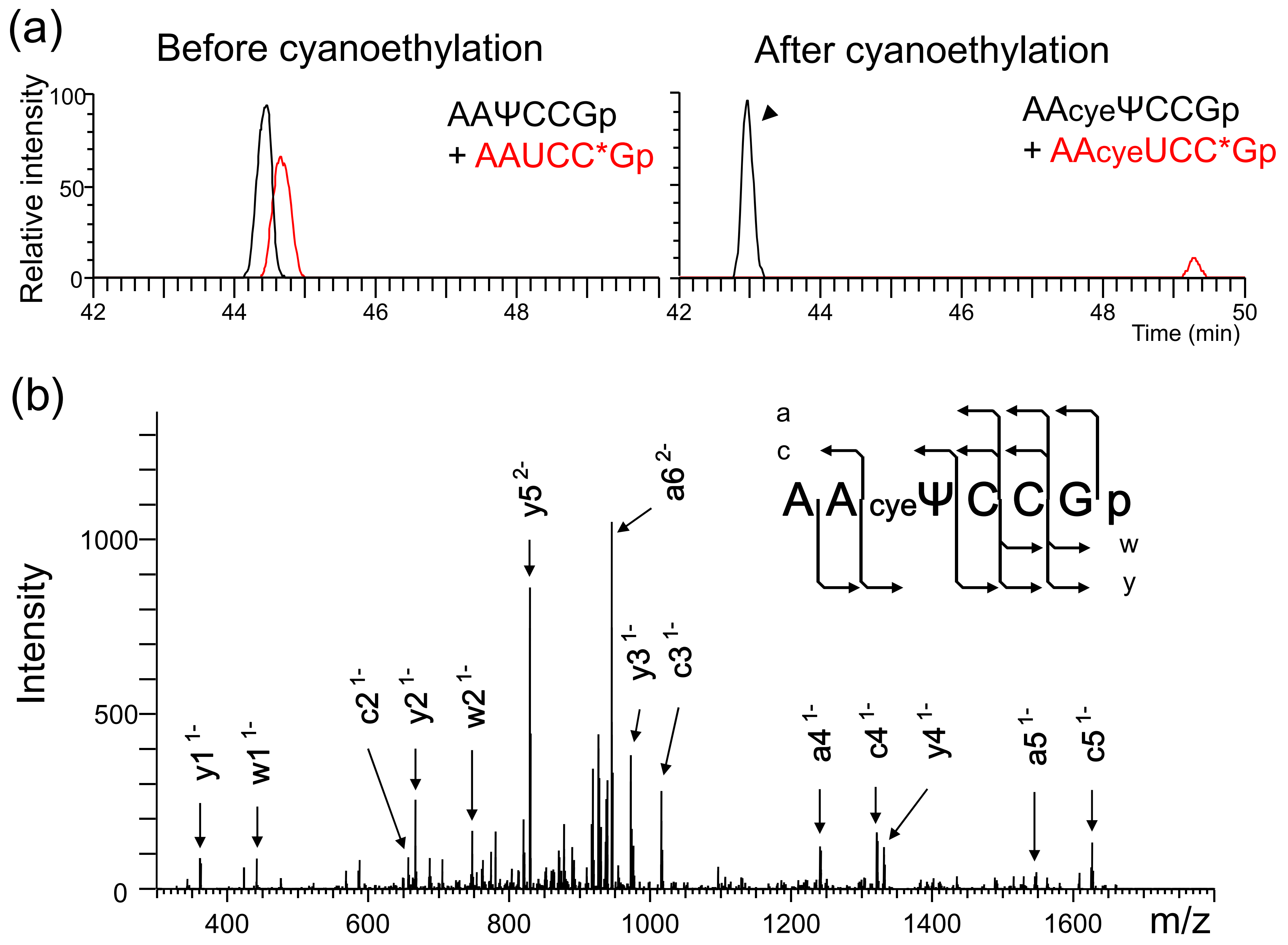

Supplementary Figure 3

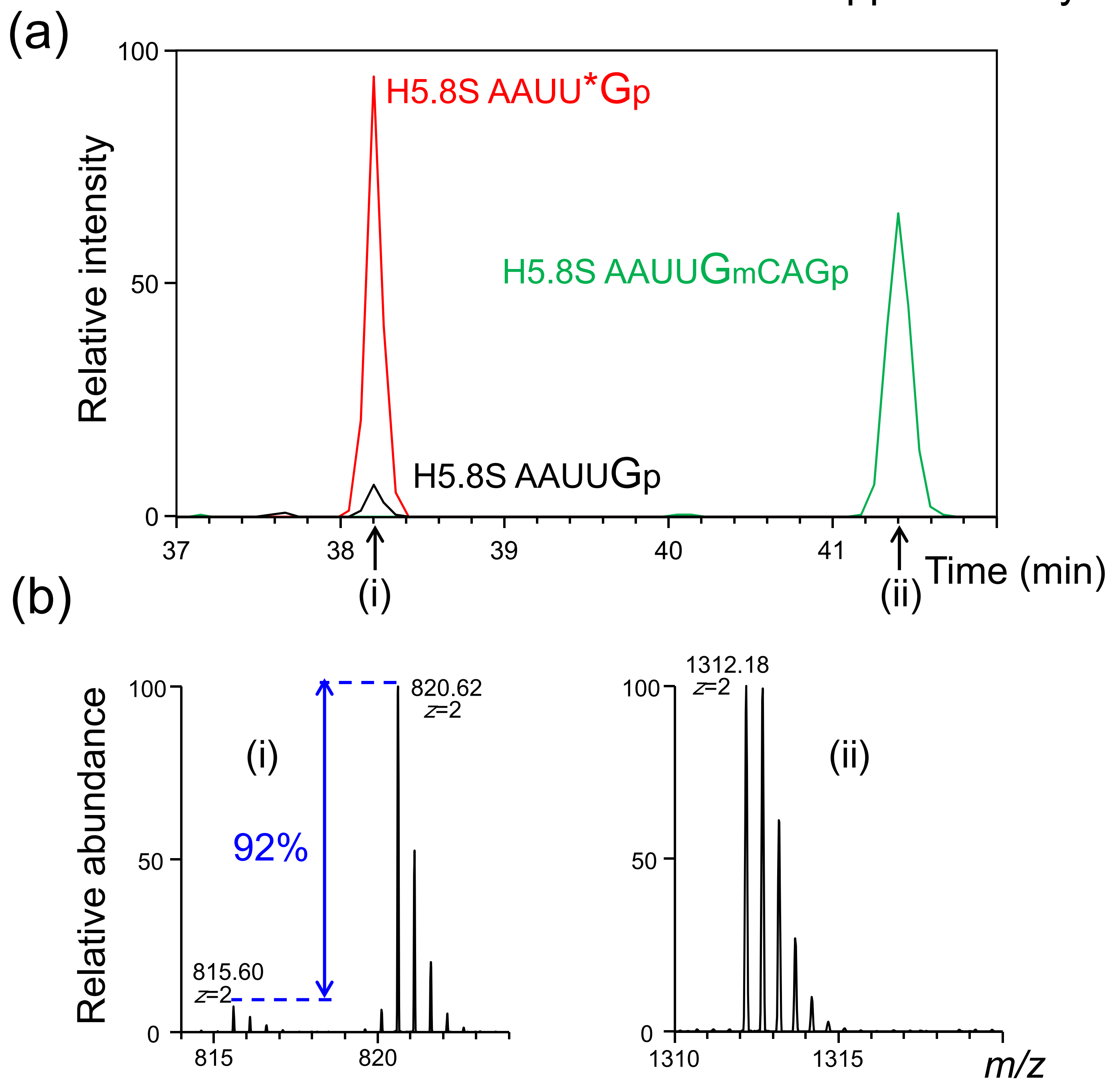

Supplementary Figure 4

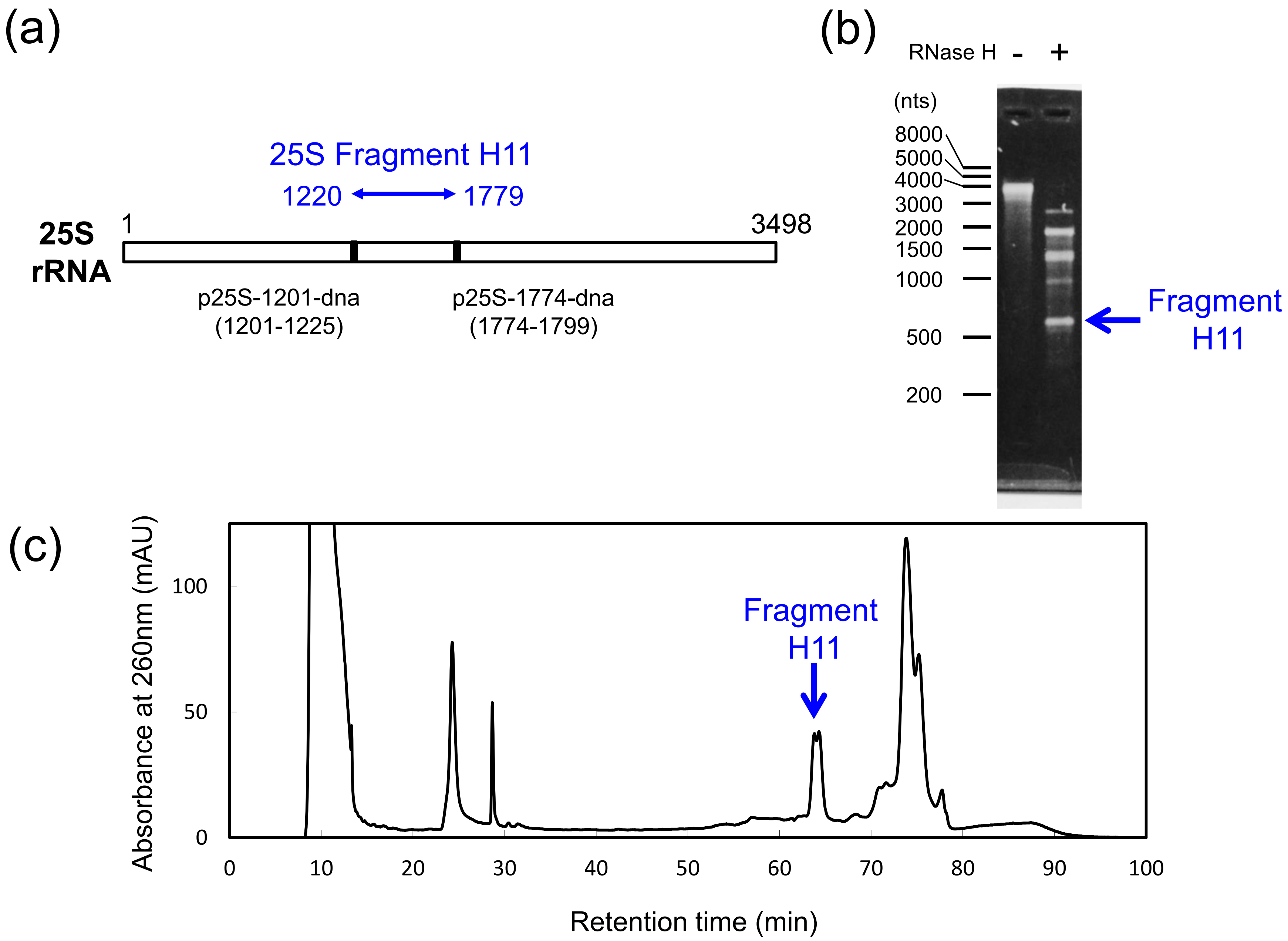

Supplementary Figure 5

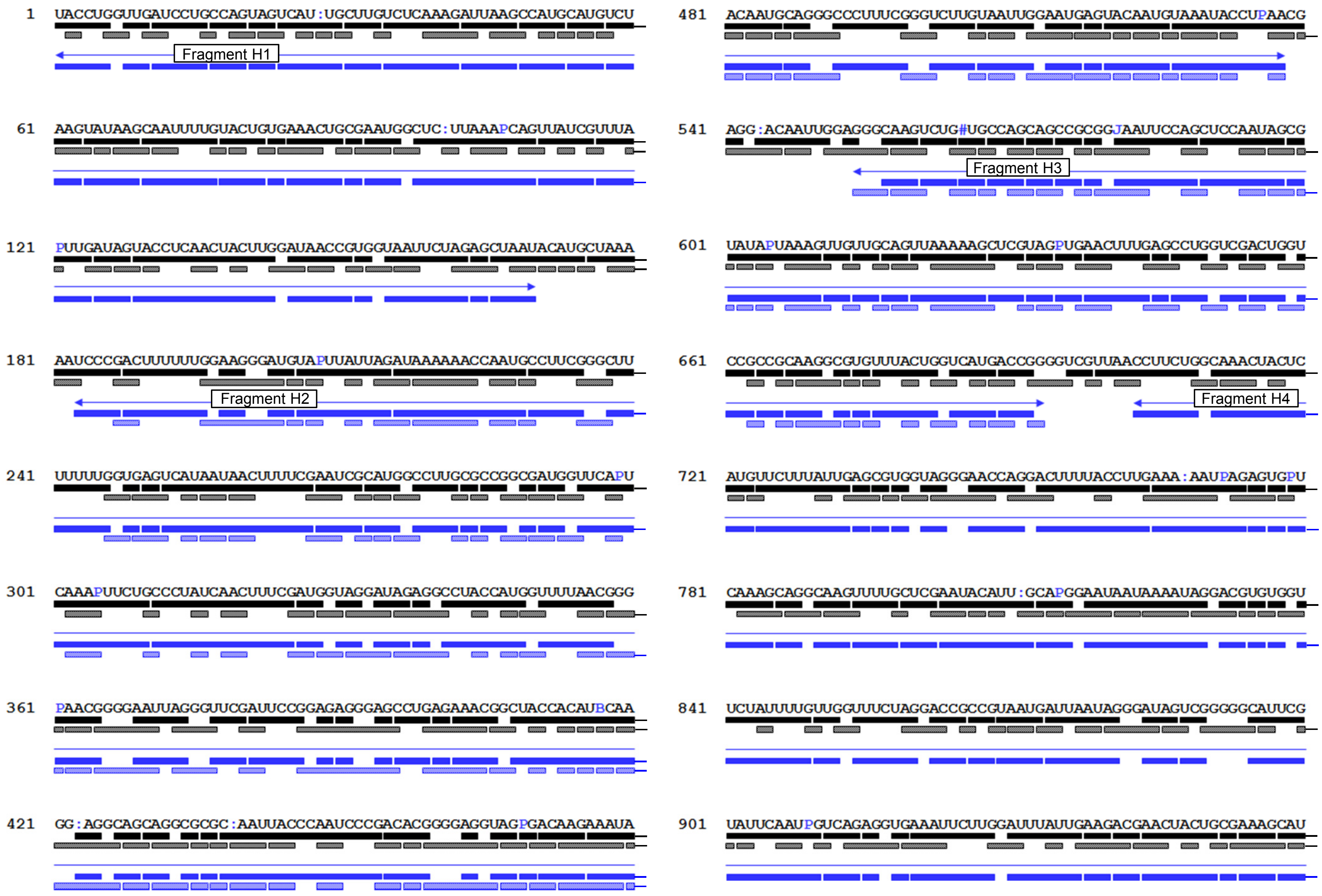

Supplementary Figure 5 -*continued*

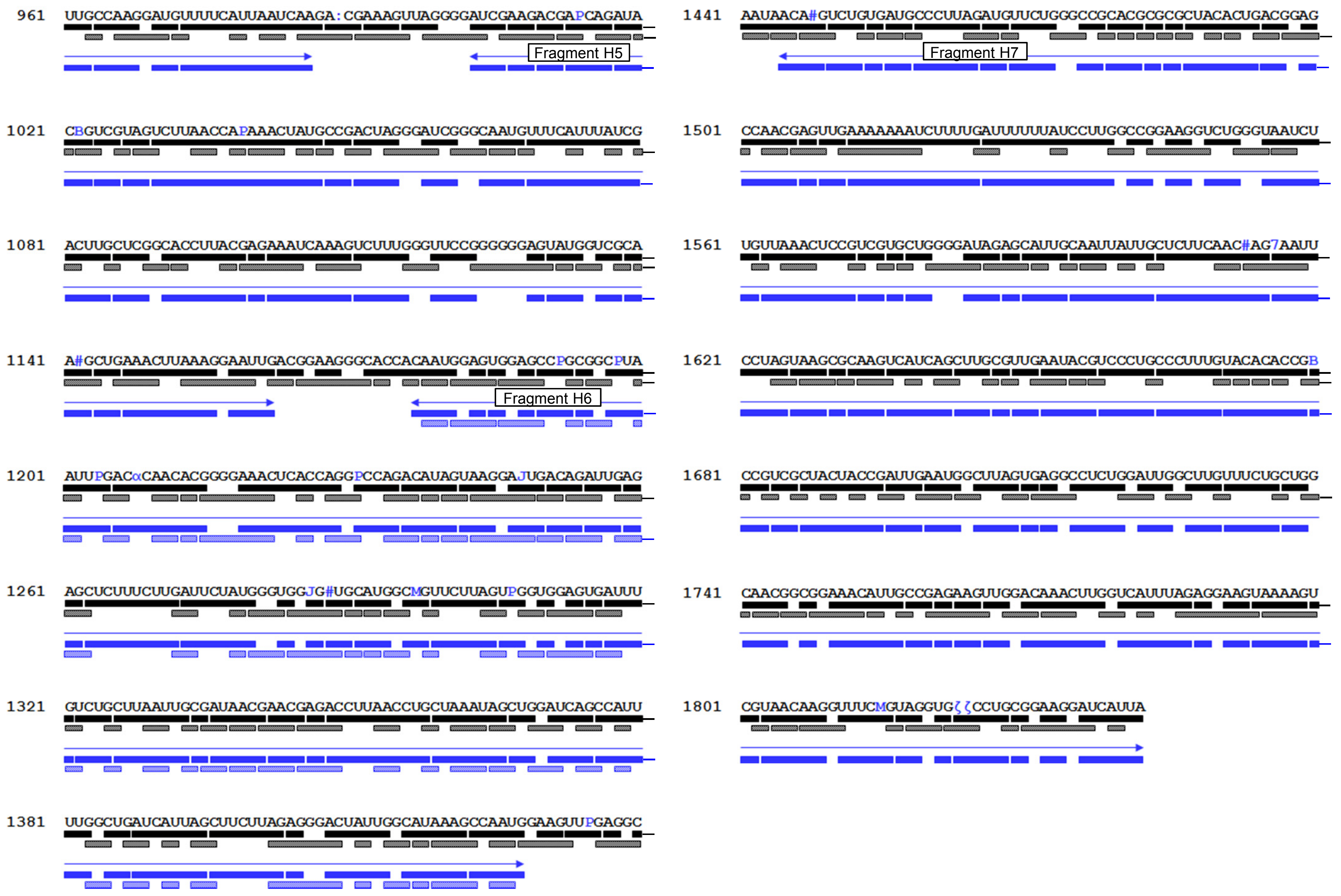

Supplementary Figure 6

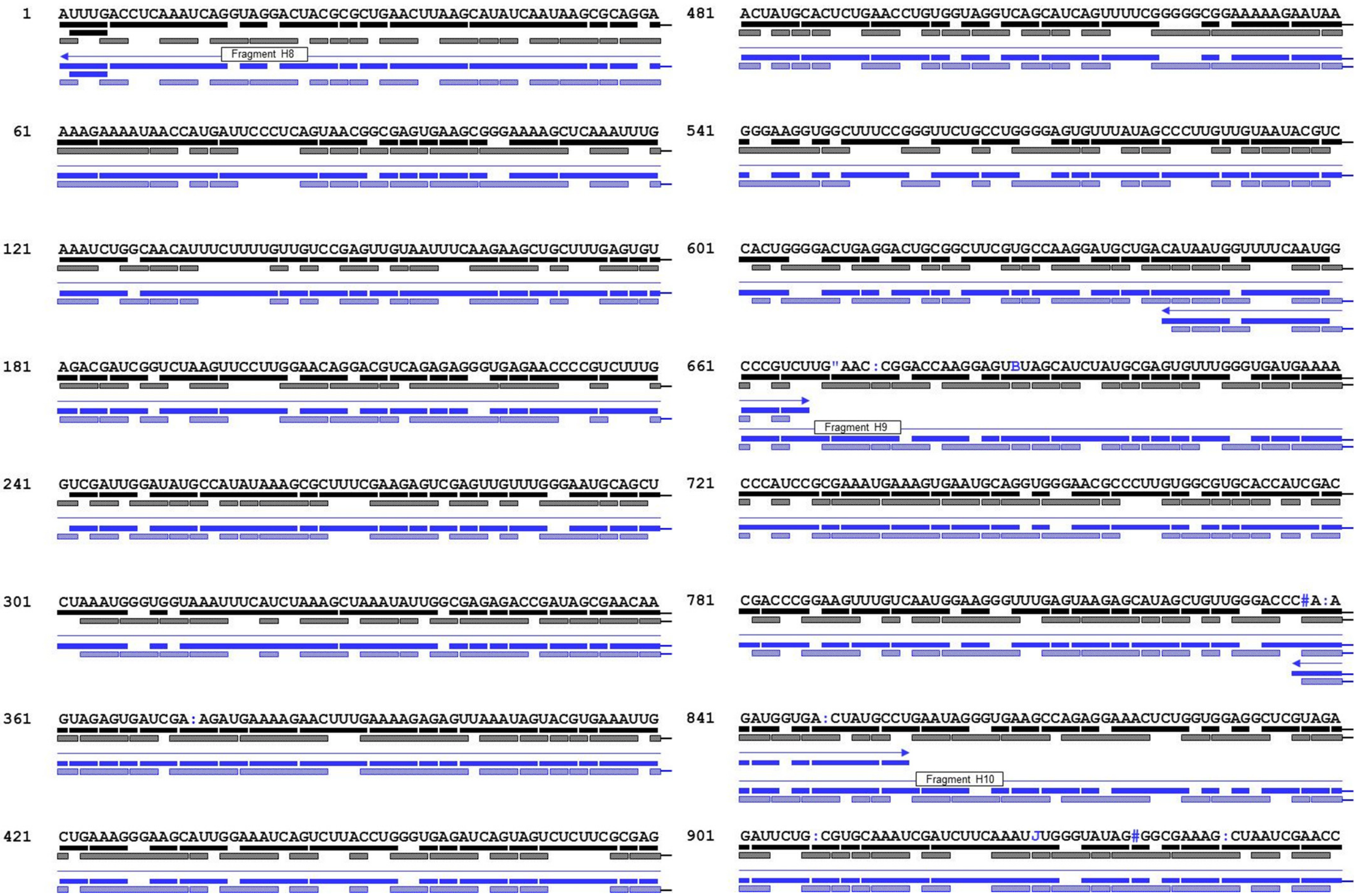

Supplementary Figure 6 -*continued*

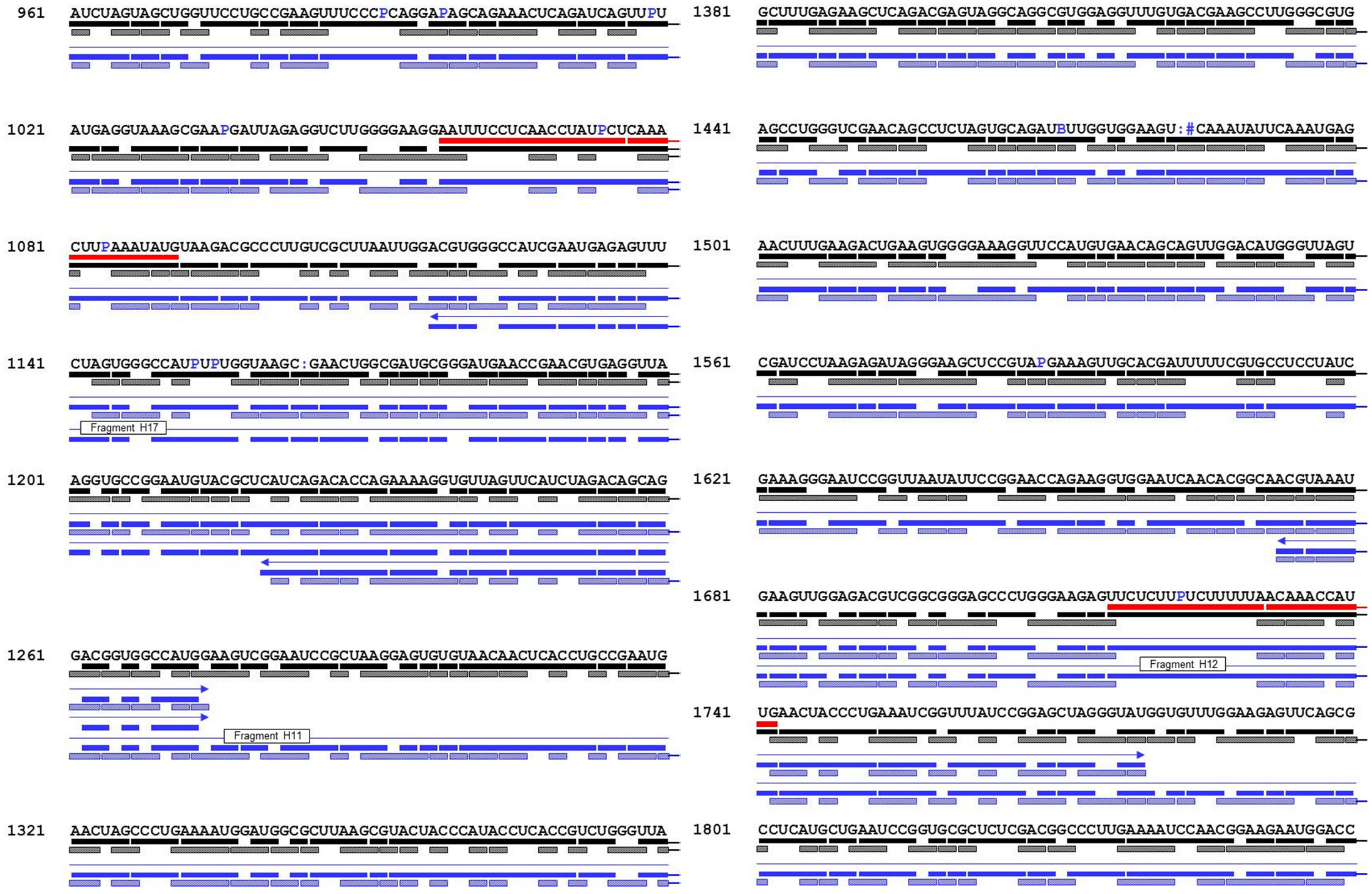

Supplementary Figure 6 -*continued*

1861 UUCGGGUCCUUGUUUUCACAUCUGGUCGUACUCAUAACCGCAGCAGGUCUCCAAGGUGAA

1921 CAGCCUCUAGUUGAUAGAACAAJGPAGAUAAGGGAAGUCGGCAAAAUGGAUCCGUAACUU

1981 CGGGAUAAGGAUUGGCUCUAAGGGUUGGGUACGUUGGGCCUUGGAACCUGAACGGUUGCU

2041 GGACUGAGCGUGGACCGAUGUCUUUUCUCGCCUUUCGGGGUGAGAAGGGAUGUUGGACCU

Fragment H13

2101 GCUUGGACCUUGGCGGCCGGGAAGUCCUUGGUCGGGCUUUUCUCCUUCUCGGGGAUUAUG

Fragment H18

2161 CUCUUACUGGCGUACGUUUAACAACCAACUUAGAACUGGUACGGACAAGGGGAAUCPGAC

2221 PGUCUAAUU"AAACAUAGCAUUGCGAUGGCCAGAAAGUGGUGUUGACGCAAUGUGAUUPC

2281 UGCCCAGUGCUCUGAAUGPCAAAGJGA:GAAAUUCAACCAAGCGCGGGUAAACGGCGGGA

2341 GPA:CPAUGACPCPCUUAAGGUAGC?AA:UGCCUC#UCAUCUAACUAGUGACGCGCAUGA

2401 APGGAUUAACGAGAUUCCCACUGUBCCUAUCUACPAPCPAGCGAAACCACAGCCUGGGGA

2461 ACGGGCCAGGCAAAAUCAGCGG#GAAAGAAGACCCUGJUGAGCPJGACJCUAGUUUGACA

2521 UUGUGAAGAGACAUAGAGGGUGUAGGAUAAGUGGGAGUAUGUUUCGGCAUACGCCGGUGA

2581 AAUACCACUACCUUUAUCGUUUCUUUACUUAAUCAAUGAAGCGGAAUUGGGAUUUAUUUC

2641 CCAUAUUCUAGCGUUAAAGUUUCUUCGCGAACUGAUCCGCGUUGAUGACAUUGUCAGGUG

Fragment H14

Supplementary Figure 6 -*continued*

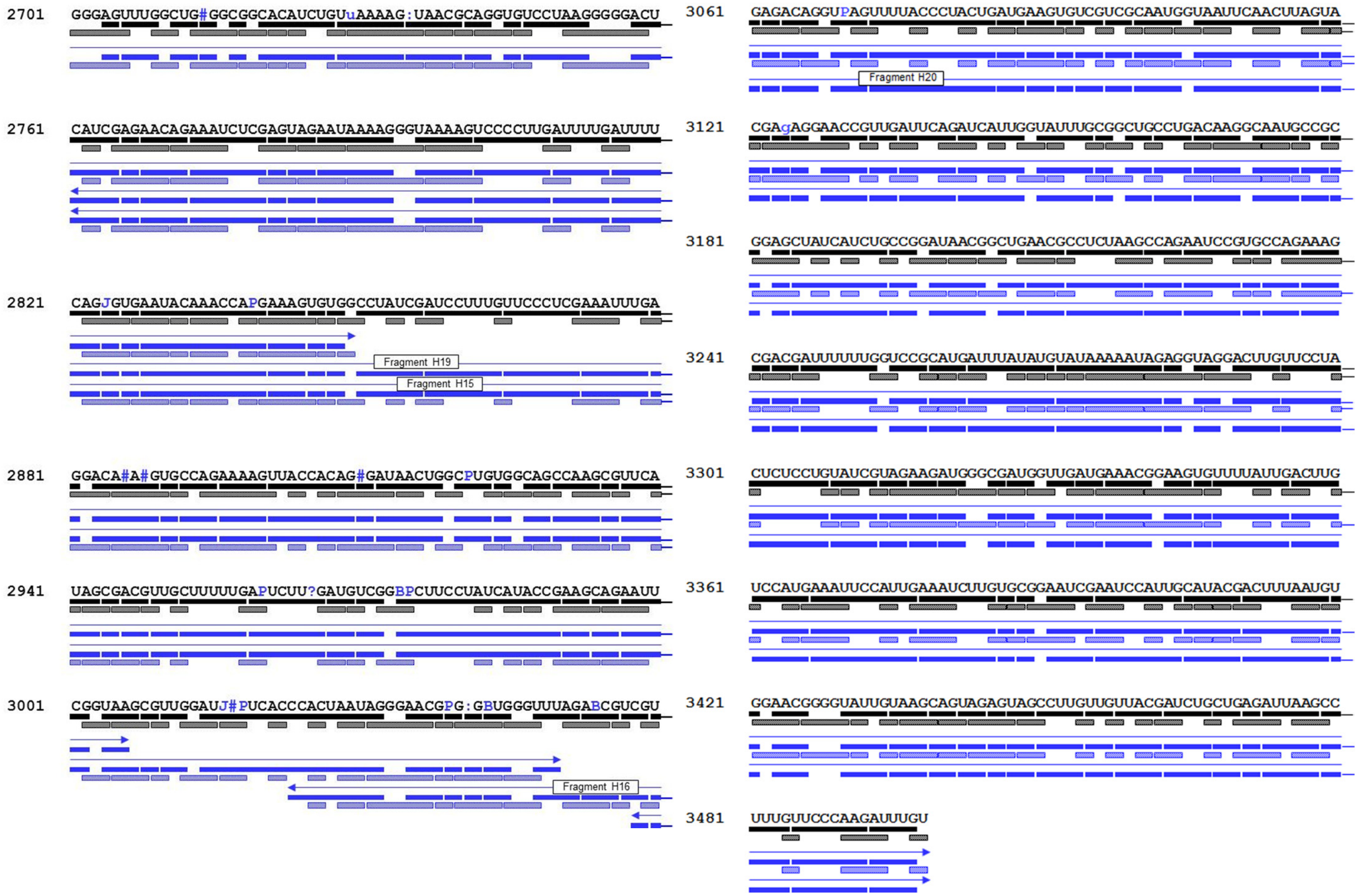

Supplementary Figure 7

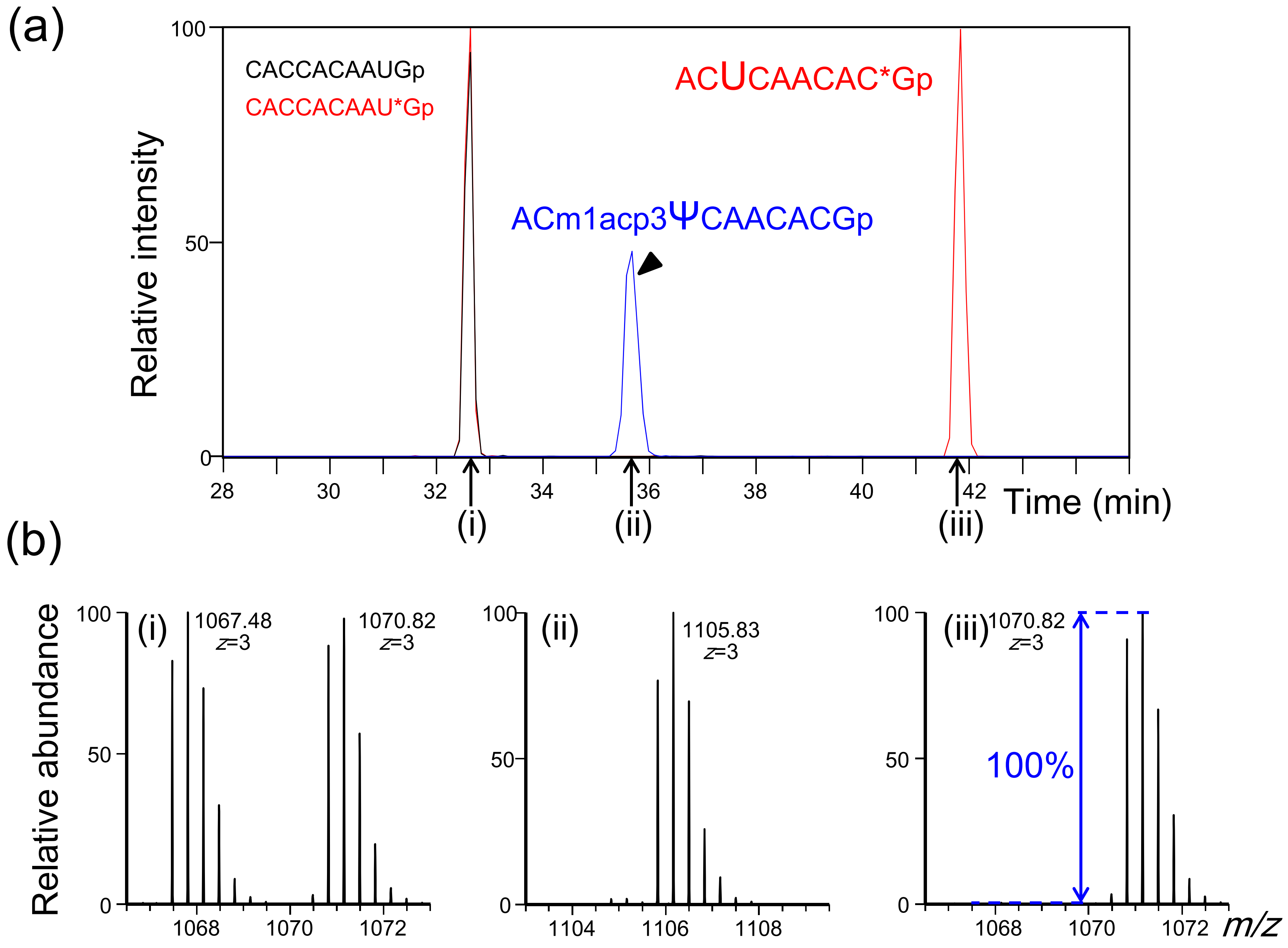

Supplementary Figure 7
-continued

(c)

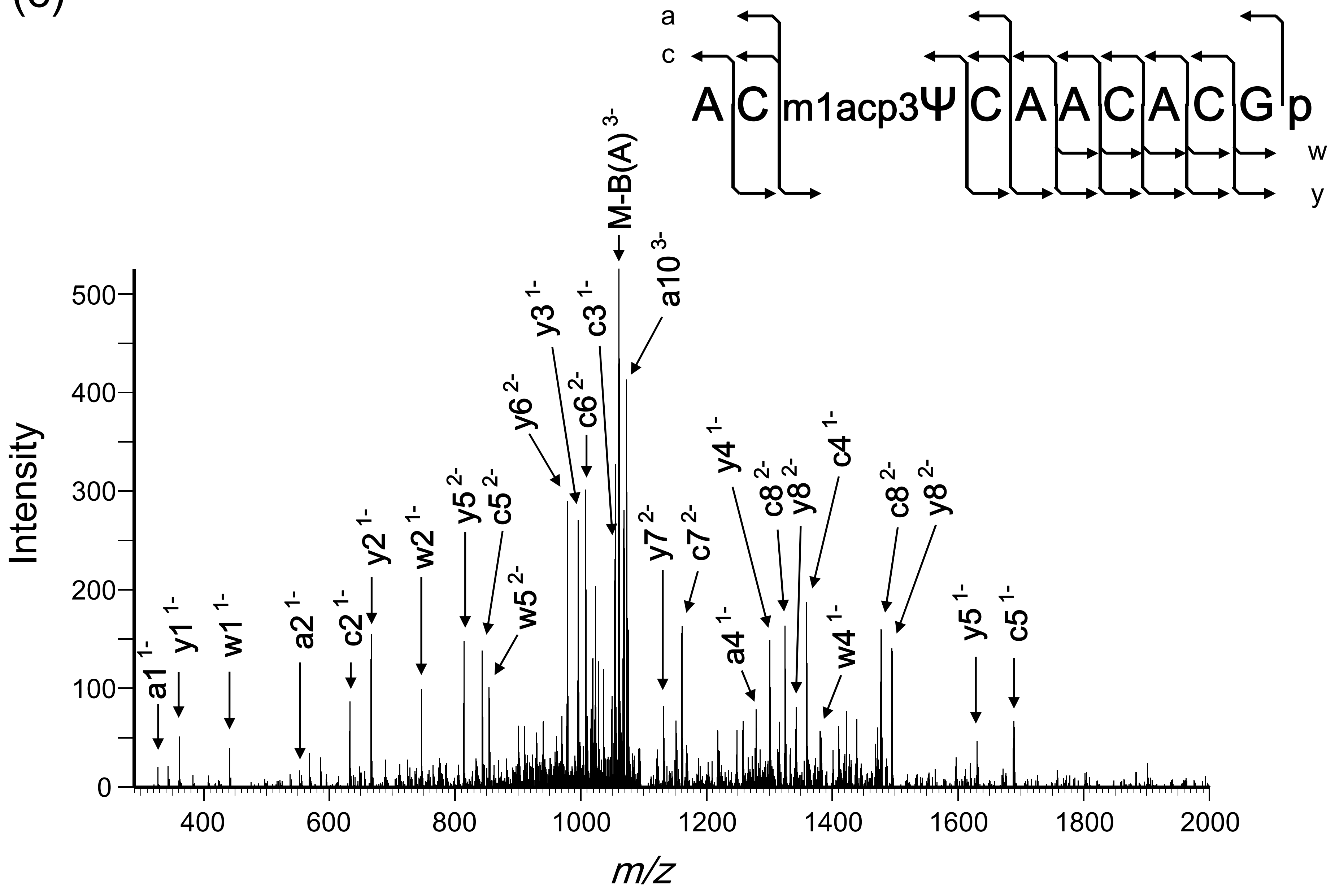

Supplementary Figure 8

A

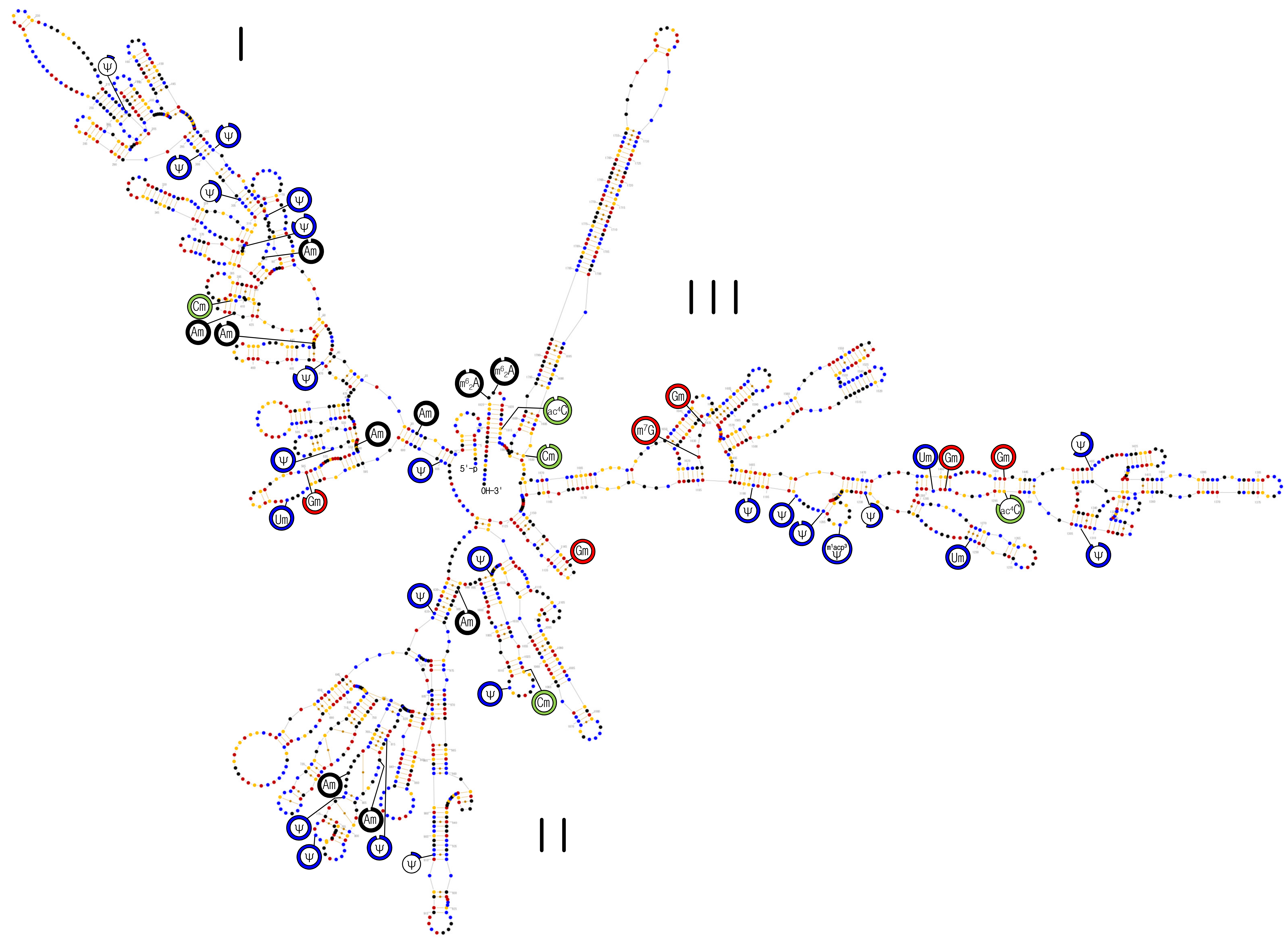

Supplementary Figure 8
- *continued*

B

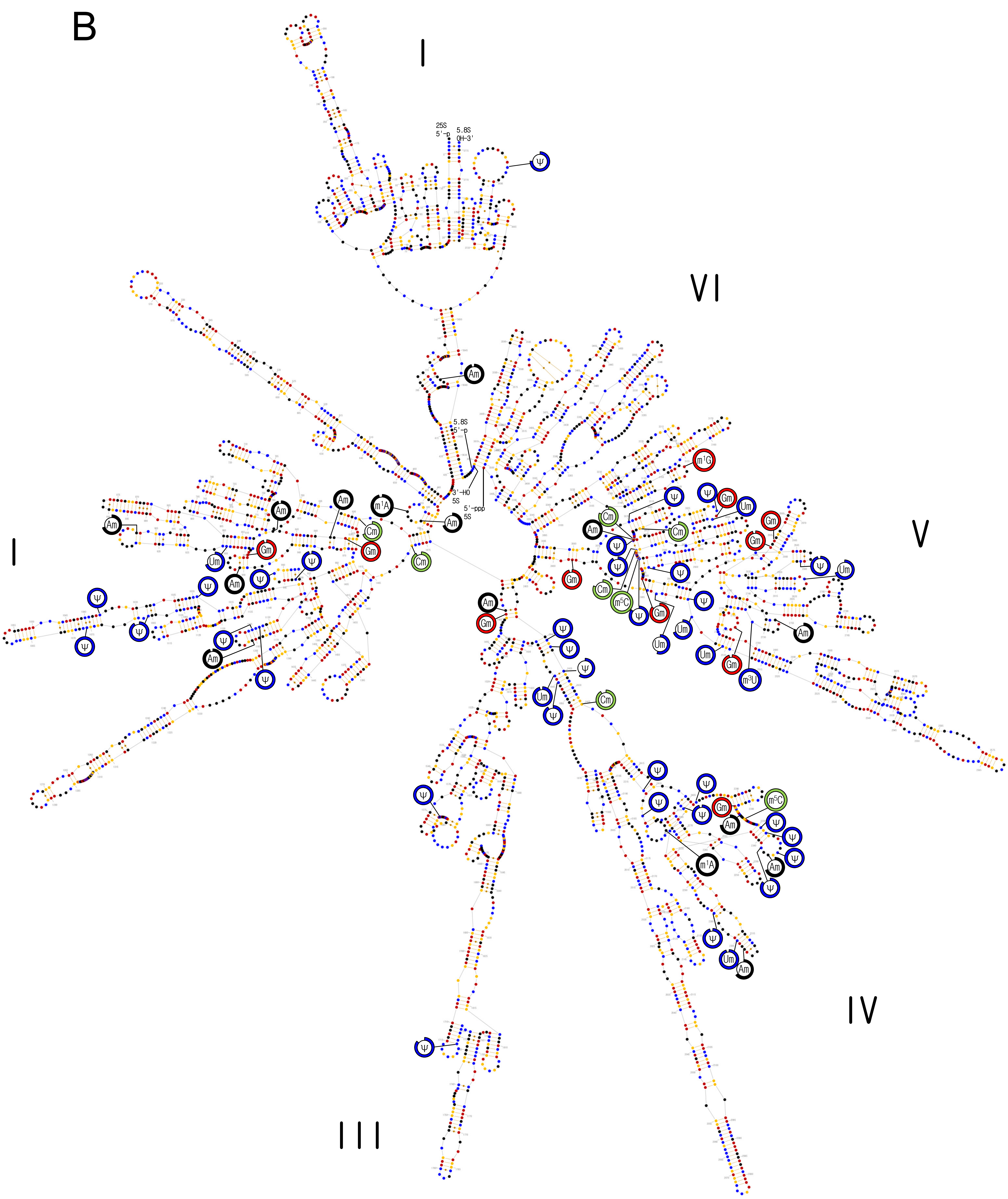

Supplementary Figure 9

A

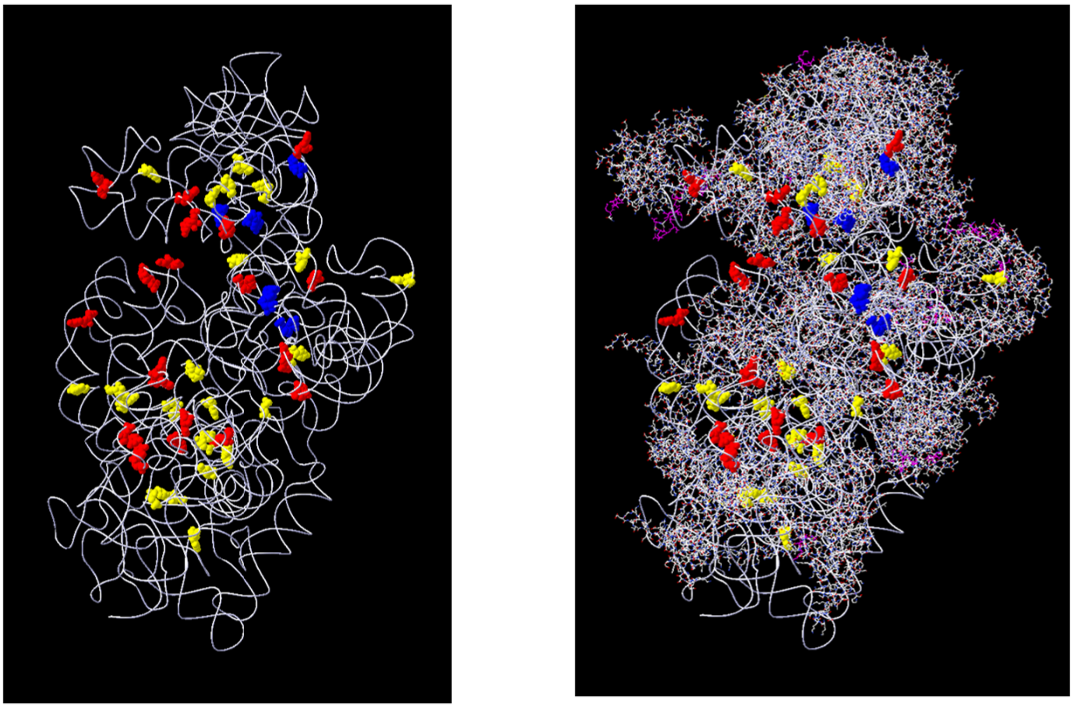

B

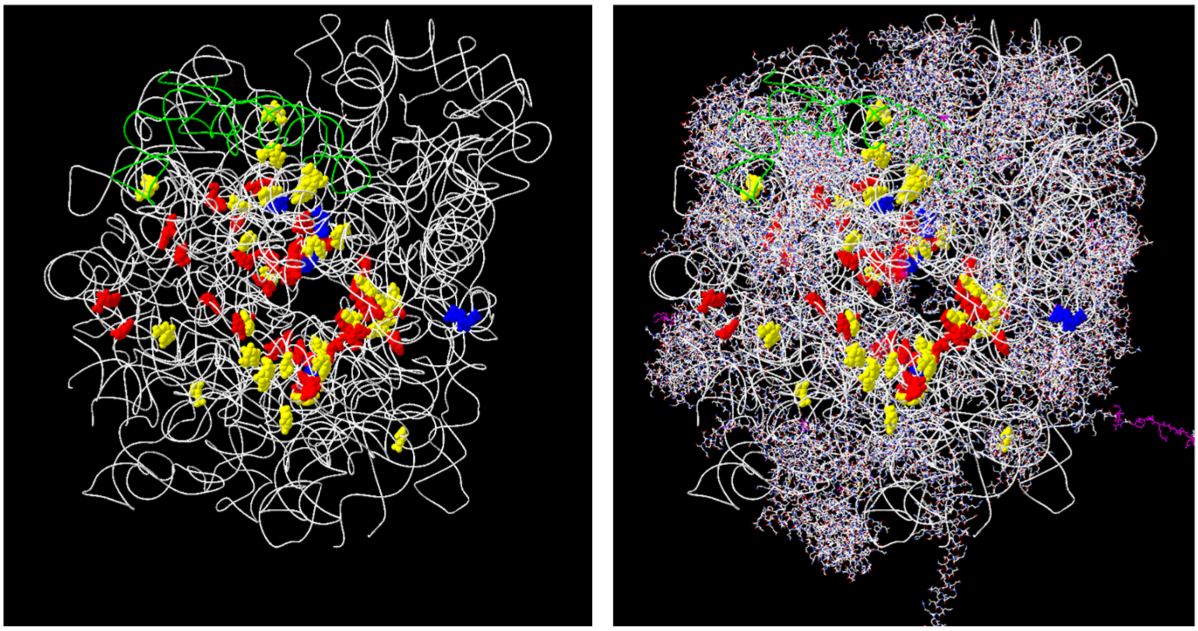

Supplement: SUPPLEMENTARY DATA [file supp_gkv560_RNA_PTM_NAR_suppl_FiguresF_Rev_150522.pdf]
